# The Electrophysiology of Basic Phrase Building

**DOI:** 10.1371/journal.pone.0158446

**Published:** 2016-10-06

**Authors:** Chris Neufeld, Stephanie E. Kramer, Natalia Lapinskaya, Christopher C. Heffner, Anton Malko, Ellen F. Lau

**Affiliations:** 1 Department of Linguistics, University of Maryland, College Park, Maryland, United States of America; 2 Department of Linguistics, Georgetown University, Washington, District of Colombia, United States of America; 3 Program in Neuroscience and Cognitive Science, University of Maryland, College Park, Maryland, United States of America; 4 Department of Hearing and Speech Sciences, University of Maryland, College Park, Maryland, United States of America; Universita degli Studi di Roma La Sapienza, ITALY

## Abstract

A defining trait of linguistic competence is the ability to combine elements into increasingly complex structures to denote, and to comprehend, a potentially infinite number of meanings. Recent magnetoencephalography (MEG) work has investigated these processes by comparing the response to nouns in combinatorial (*blue car*) and non-combinatorial (*rnsh car*) contexts. In the current study we extended this paradigm using electroencephalography (EEG) to dissociate the role of semantic content from phonological well-formedness (*yerl car*). We used event-related potential (ERP) recordings in order to better relate the observed neurophysiological correlates of basic combinatorial operations to prior ERP work on comprehension. We found that nouns in combinatorial contexts (blue car) elicited a greater centro-parietal negativity between 180-400ms, independent of the phonological well-formedness of the context word. We discuss the potential relationship between this ‘combinatorial’ effect and classic N400 effects. We also report preliminary evidence for an early anterior negative deflection immediately preceding the critical noun in combinatorial contexts, which we tentatively interpret as an electrophysiological reflex of syntactic structure initialization.

## Introduction

The past half-century of research in linguistics has demonstrated that human language is intricately structured. The prevailing view is that the unlimited expressive power of language is explained by a rich and complex set of relations between linguistic primitives: small units are combined into increasingly complex structures to denote a potentially infinite number of meanings. Since much of this structural and relational information is not explicitly present in the linguistic signal, these relationships between linguistic constituents must be reconstructed by the comprehender; without this reconstruction, a sentence would only be understood as a list of isolated words. Therefore, the task of language comprehension can be broadly construed as one of structure-building.

The goal of the current study is to isolate the neurophysiological correlates of the basic combinatorial processes required for comprehension of simple two-word noun phrases (*blue car*). Much of the vast literature on the electrophysiology of language comprehension examines the processing of relatively complex stimuli—typically full sentences—replete with rich lexical syntactic, and sometimes prosodic detail. However, since full sentences require many combinatorial processes, designs that compare the responses to different sentences do not contrast the presence or absence of combinatorial operations but rather vary in whether a particular operation is thought to occur in one sentence and not the other. Here, we follow recent fMRI and MEG work arguing that two-word phrases provide a unique opportunity to examine the minimal contrast between an unstructured sequence that does not require combinatorial operations and a structured sequence that does [1–3]. By using EEG, here we emphasize the time-course of structure-building and explore the relationship between these ‘structure-building’ responses and the ERP components that have been previously investigated in the literature on the electrophysiology of language.

### Background

In language comprehension we perceive relations between units at various levels, among them phonology, syntax and semantics. One aim of neurolinguistics is to isolate the processes responsible for each level of perception. In comprehending *rusty car*, for instance, we perceive both that *rusty* and *car* are related syntactically as an adjective adjoined to a noun, and also that its meaning conjoins the properties expressed by its parts: roughly it means ‘rusty and car’. The two sorts of relations are distinct and dissociable. Adverbs cannot combine with nouns, for example, even if the result would express a good combination of concepts: *rustily car* is illicit, perhaps only for reasons of syntax. How the brain represents these temporary syntactic and semantic relationships in memory during the course of sentence comprehension is a critical question that is not yet well-understood.

One central paradigm for exploring this question has been to compare the processing of word sequences that can be structured into sentences with the processing of word sequences that cannot (scrambled sentences or word lists). This paradigm has been most commonly used in fMRI, where the low temporal resolution of the signal makes comparisons across multi-word sequences less straightforward. These studies have reported increased activity for structured relative to unstructured sequences in a number of regions including parts of anterior temporal cortex, inferior frontal cortex, and angular gyrus [4–12]. These whole-sequence comparisons have less often been done in EEG or MEG, but Van Petten and Kutas [13] reported a sustained positive shift across the course of the word sequence for unstructured relative to structured conditions in EEG. Using MEG, Brennan and Pylkkänen [14] report increased activity in temporal, inferior frontal, and ventromedial prefrontal cortices in the response to open class words in structured vs. unstructured sequences. However, while these studies have been useful in locating a set of candidate regions for encoding and manipulating syntactic and semantic relations, the relatively large number of syntactic and semantic computations that occur over the course of a sentence relative to a word list make it difficult to associate particular regions or time-windows with specific computations in this paradigm. A recent series of MEG experiments by Bemis and Pylkkänen have aimed to isolate a few of the most basic combinatorial operations through simpler stimulus comparisons that are centered around noun-adjective combination [1, 15–18], with the first of these studies [1] providing the basis for our current investigation. Bemis and Pylkkämen [1] contrasted brain activity when two consecutive stimuli could be composed into a phrase (*red boat*) with brain activity when such combination was either not possible (*xkq boat*), or discouraged by the task (*cup, boat* in a word list task). They reported that left and right anterior temporal lobes (LATL, RATL) and ventromedial prefrontal cortex (vmPFC) showed an increased response to nouns in combinatorial contexts (*red boat*) over all other conditions, with the ATL response earlier (184-255ms post-stimulus), and the vmPFC response later (331-480ms). Based on earlier MEG studies from their group implicating vmPFC in semantic processing and on the assumption that syntactic relations temporally precede semantic ones, Bemis and Pylkkänen [1] tentatively attributed LATL activity to syntactic combination and vmPFC activity to basic semantic composition. This perspective has been partially supported by subsequent replication of the early combinatorial ATL effect in both visual and auditory modalities [17] and the failure to observe similar ATL effects for mathematical or pictorial combination [16]. However, later work by this group has raised some doubts about the location of the later combination effect as well as the interpretation of the earlier ATL effect. Bemis and Pylkkänen [17] failed to replicate the vmPFC effect, finding instead a combinatorial effect in angular gyrus in a similar (336-390ms) time-window; and several subsequent studies by Pylkkänen and colleagues [19, 20] have led them to argue that ATL activation reflects processing associated with conceptual or semantic combination.

### Current study

In the current study we examined ERPs in both the early time-window in which Bemis and Pylkkänen [1] reported combinatorial activity in left ATL, and in the subsequent time-window in which differential combinatorial activity was localized to various regions across previous work. Although to our knowledge no prior EEG studies have examined a similar contrast, the time window in which combinatorial vmPFC activity is reported in Bemis and Pylkkänen [1] overlaps with that of the N400, a well-studied ERP component observed in response to meaningful stimuli [21]. Amplitude modulations of the N400 have been broadly interpreted to index aspects of semantic integration or contextually modulated lexical retrieval. While N400 effects have most commonly been localized to left-lateralized anterior and posterior temporal cortex [22, 23] and not vmPFC, Bemis and Pylkkänen’s [1] data does indicate a subthreshold effect of combination in a left anterior temporal ROI between 300-400ms, and subsequent work from this group (e.g., [19, 20]) has argued that activity in this area and time window is specifically associated with semantic or conceptual combination. Therefore, one question that the current ERP study was designed to assess was the extent to which the combinatorial effects observed by Bemis and Pylkkänen resemble the N400 effects observed elsewhere in ERP findings.

We replicated the basic structure of the MEG experiment described in Bemis and Pylkkänen [1]. We predicted that nouns presented in immediate compositional contexts and those that are not (e.g., *blue car* and *rnsh car*) would elicit different electrophysiological responses. We similarly expected that this difference would not extend to two words presented in a list task not requiring combination but still requiring attention to both stimuli (i.e., *blue car* and *lamp car* would not elicit similar responses). Our aim was identifying the temporal and spatial characteristics of effects of basic combination in ERP, and comparing these effects to well-established ERP responses to language comprehension such as the N400 effect.

We extended Bemis and Pylkkänen’s [1] design to include an additional pseudoword control condition (e.g., *yerl car*). This additional contrast was designed to ensure that any neurophysiological differences found between nouns in compositional contexts (e.g., *blue car*) and non-compositional contexts (e.g., *rnsh car*) were not solely due to phonological viability of the first word or the prosodic viability of the phrase. Complex noun phrases have a prosodic structure that could be implicitly constructed in reading, as has been demonstrated in self-paced reading designs for more complex phrases [24]. However, in the nonword condition no such prosodic composition is possible, since consonant strings have no associated phonology, and thus, presumably no phonological structure greater than the word-level can be created. Thus, differences between *blue car* and *rnsh car* could be at least partially driven by prosodic processing rather than syntactic or semantic combination. The pseudoword contexts in the *yerl car* condition were pronounceable and therefore differences between *blue car* and *yerl car* are more likely attributable to syntactic or semantic combinatorial processing.

As indicated above, our analyses largely focused on the same time-windows as Bemis and Pylkkänen [1]. However, one different analysis choice that we made in the current study was to extract a large epoch including both adjective and noun and to then baseline the resulting event-related potentials to the time-window immediately preceding the context word that started the trial, rather than baselining them to the time-window immediately preceding the critical noun. We did this in order to ensure that any differences we observed immediately following the noun could not be attributed to different context words eliciting different responses immediately prior to the noun and contaminating the baseline.

Finally, given the partial overlap in timecourse between the prior MEG results and the N400 effect observed in ERP, we also ran a simple N400 effect localizer in a subset of participants in order to directly compare the timing and topography of the effect elicited by combinatorial activity with that of classic N400 context effects. We used a predictability manipulation with adjective-noun sequences that has previously been shown to elicit large N400 effects (nouns highly predicted by the preceding adjective demonstrate reduced N400 amplitudes relative to those that are not; see [25]).

## Methods

### Materials

The experiment used a 2 × 3 within-subjects design, crossing two blocked tasks (composition task and list task) and three within-block stimulus item conditions (real words, nonwords, and pseudowords). As one of our primary goals was to relate the effects previously observed in MEG to effects in EEG, here we closely follow the design and procedure of Bemis and Pylkkänen [1]. In both tasks, participants were presented with two orthographic stimuli in succession followed by a picture of a colored object. The second stimulus was always a monosyllabic concrete noun (e.g., *car*), while the initial stimulus varied across conditions. In the pseudoword condition, the first stimulus was a phonologically viable nonword string (e.g., *yerl*). In the nonword condition, the first stimulus was a consonant string (e.g., *rnsh*). The only difference in stimuli across tasks was in the real word condition, which was an adjective in the compose task (e.g., *blue*) and a noun in the list task (e.g., *lamp*). An example item set is illustrated in Table 1.

**Table 1:** Example stimulus set for the main experiment.

Condition	Compose Task	List Task
Real word	blue car	lamp car
Psudoword	yerl car	yerl car
Nonword	rnsh car	rnsh car

In both tasks, participants had to assess the match between the image and the preceding stimuli, but the tasks differed in what criteria participants were instructed to use for evaluating the match. In the compose task, participants were instructed that the final picture should only be considered a match if it depicted the noun of that color (e.g., *blue car* followed by a picture of a blue car was considered a match, but a blue cup or a red car was not considered a match). In the list task, participants were instructed that the final picture should be considered a match if it depicted either of the words (e.g., *lamp car* followed by a picture of either a car or a lamp was considered a match). For both tasks, participants were instructed that nonword and pseudoword trials should be considered a match if the picture matched the preceding noun (e.g., *rnsh car* or *yerl car* followed by a picture of a car of any color was considered a match).

The task manipulation is designed to control for the lexical status of the first word in the compose task. Any amplitude differences found between the real word condition and the other conditions in the compose task (e.g., *blue car* vs. *rnsh car*) could plausibly be due to the status of *blue* as a real word compared to *rnsh*. Examining the interaction between condition and task, then, makes it possible to factor out the effects of the lexical status of the first word on any electrophysiological findings. While the processing of adjectives and nouns is also not identical, we followed Bemis and Pylkkänen in using nouns instead of adjectives in the list task because in contrast to noun sequences, which can naturally be perceived as lists, composition of an adjective followed by a noun may be too automatic to be prevented by instruction.

### Materials

Color adjectives, nouns, and images were taken from Bemis and Pylkkänen’s [1] study, and pseudowords and nonwords were created for the current study. For the real word condition in the compose task, the first stimulus was one of six color adjectives (*red, blue, pink, black, green, brown*). For the real word condition in the list task, the first stimulus was one of six length-matched nouns (*cup, boat, lamp, plane, cross, house*). For the pseudoword and nonword conditions in both tasks, stimuli were one of six randomly combined sets of letters matched in length and bigram frequency to the color adjectives (pseudoword: *til, frie, yerl, spett, clior, twamm*; nonword: *nts, rnsh, lthr, ttrsp, htsth, ksplt*). Two of these pseudowords would likely be pronounced identically to real-words: *til* would be pronounced identically to *‘til* or *till* and *frie* would likely be pronounced identically to either *fry* or *free*. We discuss this briefly in our results. For the second stimuli in all conditions in all tasks, the word was one of 25 monosyllabic nouns (*disc, plane, bag, lock, cane, hand, key, shoe, bone, square, bell, boat, bow, car, cross, cup, flag, fork, heart, lamp, leaf, note, star, tree, house*).

Ten different randomized lists were created. Each list contained an equal number of trials (300 each) in the list and compose task, and lists were counterbalanced for the order of the two tasks. Within each task, each of the three conditions contained 100 trials, of which 50 were match and 50 were mismatch trials. For the compose task, the real word-condition had an additional division of the mismatch trials: 25 were a mismatch on the object color (e.g., a blue car paired with pink car) and 25 were a mismatch on the identity of the noun (e.g., a blue car paired with blue cane). Note that because the critical word preceded the matching or mismatching image, the match factor was irrelevant for analysis of the critical ERPs, such that 100 trials were available for analysis for each condition and participant. Participants responded to match trials by pressing the J key on their keyboard and mismatch trials by pressing F. The order of each task was counterbalanced across participants, and the order of items within each block was randomized.

### Procedure

**Fig 1:**
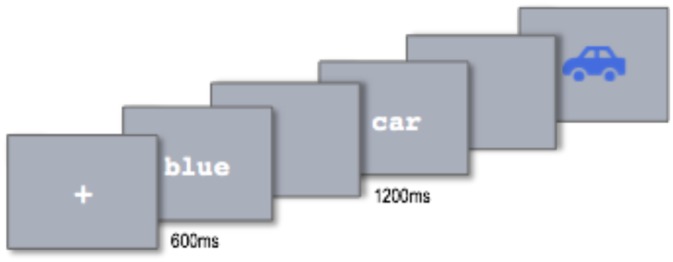
Illustration of the stimulus presentation sequence in a single trial. Each trial began with a fixation cross, followed by 300 ms of blank screen, 300 ms of word 1, 300 ms of blank screen, 300 ms of word 2, 300 ms of blank screen, and the image.

Participants were seated approximately 15” from the presentation screen. Each session was divided into two blocks—one for each task—with a practice session in the beginning of each block and a break in between. Directions and stimuli were visually presented in the center of the screen in 12-point white Courier font centered on a grey background. The trial structure is illustrated in Fig 1. Each trial began with a fixation cross. Each of the words was presented for 300 ms, followed by 300 ms of blank screen. The image at the end of each trial was displayed until the subject accepted or rejected it as a match for the preceding words. Trials were separated by a variable inter-stimulus interval following a uniform distribution, with an average of 400 ms, ±100ms jitter.

### N400 Localizer

Following the main experiment, a subset of participants completed a short second experiment designed to elicit a standard N400 predictability effect through contrasting high- and low-predictability noun-adjective pairs. 120 high-probability (predictable) adjective-noun pairs were created by combining a highly-constraining adjective extracted from the Corpus of Contemporary American English (COCA; [26]) with the noun that most frequently follows it such that the probability of encountering the noun given the adjective was greater than 50%. 120 matching low probability (plausible) adjective-noun pairs were created by pairing the same nouns with weakly-constraining adjectives, also extracted from COCA, such that the probability of encountering the noun given the adjective was less than 2% and the probability of encountering any one noun given the adjective was less than 15%.

1. High-Probability (predicted condition): *runny* *nose*
2. Low-Probability (control condition) *dainty* *nose*

Items were divided equally into two randomized stimuli lists of 60 high-probability and 60 low-probability bigrams in a Latin Square design such that no words were repeated on a given list and each participant saw only one list. Stimuli were presented on a computer monitor in white 24-point case Arial font on a black background. Each trial began with a fixation cross at the center of the screen for 700 ms, followed by a 200 ms blank screen. The adjective was then presented for 500 ms, followed by a 100 ms blank screen, and the noun was presented for 900 ms, followed by another 100 ms blank screen. In order to ensure attention to the stimuli, participants were informed that they would be asked to perform a memory recall test at the end of the experiment that would require them to distinguish seen adjective-noun pairs from unseen (rearranged) pairs.

### Participants

33 total students and affiliates of the University of Maryland (14 male) participated in the study. Of these, 20 participated in both the main experiment and the N400 localizer experiment, while 13 participated only in the main experiment. Seven of the main experiment datasets were excluded due to excessive artifacts (> 45% trials) and seven were excluded due to low accuracy on the picture matching task (<70% correct for any given condition), leaving data from 19 participants (10 male, mean age 22.2). In the majority of these exclusion cases, participants were confused about the criteria for the compose task, where the criteria for a ‘match’ response took into account both words in the two-word condition but only the second word in the pseudoword and nonword conditions. Six of the N400 localizer participants were excluded due to excessive artifacts, leaving localizer data from 12 participants (6 male, mean age 21.1) for analysis. All participants gave prior written consent according to established guidelines of the Institutional Review Board of the University of Maryland and received monetary compensation for their participation. All participants were between the ages of 18-40, were native speakers of American English, and were right-handed as assessed by the Edinburgh Handedness Inventory [27]. This study was approved by the Institutional Review Board of the University of Maryland.

### Electrophysiological Recording

Twenty-nine tin electrodes were held in place on the scalp by an elastic cap [28] in a 10-20 configuration (O1, Oz, O2, P7, P3, Pz, P4, P8, TP7, Cp3, CPz, CP4, TP8, T7, C3, Cz, C4, T8, FT7, FC3, FCz, FC4, FT8, F7, F3, Fz, F4, F8, FP1). Bipolar electrodes were placed above and below the left eye and at the outer canthus of the right and left eyes to monitor vertical and horizontal eye movements. Additional electrodes were placed over the left and right mastoids. Scalp electrodes were referenced online to the left mastoid and re-referenced offline to the average of left and right mastoids. Impedances were maintained at less than 15 kΩ. The EEG signal was amplified by a NeuroScan SynAmps^®^ Model 5083 [29] and was continuously sampled at 500 Hz by an analog-to-digital converter with an 0.05-100Hz online bandpass filter.

### Analysis

We examined ERP amplitudes that corresponded to activity occurring after the onset of the second word, as well as changes that reflect preparatory processes during or after the onset of the first word. For each trial, we extracted a single epoch that was timelocked to the onset of the second word in the trial (the critical word), but extended to cover the response to the preceding word (presented 600ms earlier) as well. We conducted analyses on mean ERP amplitudes for four time-windows within a resulting -650:600ms epoch, ranging from the 50ms prior to the onset of the first word (at -600ms) to the beginning of the response window following the critical second word. A low-pass finite impulse response filter at 40Hz was applied after epoching to the EEG timeseries for both experiments. ERPs were baselined to activity in the 50ms immediately preceding the first stimulus of the trial, that is, a period from -650 to -600 ms relative to the critical stimulus. This period was chosen because the first stimulus was one of three different conditions (real word, pseudoword, or nonword)—each of which might have had a different ERP prior to the (second) critical stimulus. Epochs containing muscular or ocular artifacts were excluded from analysis. Rejections were made according to peak-to-peak difference (default rejection threshold of 100 μV). Ocular movement rejections were made based on VEOG/HEOG electrode amplitude (default rejection thresholds of 40 and 25 μV, respectively). Automatic artifact rejection was then manually verified. Three datasets had channels that were excessively noisy, which were removed from the dataset and interpolated on the basis of surrounding channels. On average, 23.5% of epochs were excluded per participant.

We examined four time windows in our analyses, based on previously published work or on visual inspection. We examined the 184:256ms post-critical stimulus time-window in which Bemis and Pylkkänen [1] reported significant combinatorial effects in ATL. We also assessed effects during the 300:400ms time-window for both the initial word of the trial and the second (critical) word of the trial by analyzing the 300:400ms time-window following the onset of each word, where the N400 appeared to be at its maximum and which roughly overlapped with the time-window in which Bemis and Pylkkänen [1, 17] reported combinatorial effects in vmPFC and AG. We were interested in the N400 response to the context word because previous work has demonstrated differential N400 amplitudes for pseudowords, nonwords, and real words depending on experimental context [30–32]. Finally, as visual inspection indicated the presence of differences immediately prior and subsequent to presentation of the critical word, we conducted analyses on the -50:100ms time-window to assess the reliability of these differences.

In each time-window, we conducted an omnibus repeated-measures 3 × 2 × 2 ANOVA (condition × task × anteriority) on a subset of 20 electrodes (anterior: F7, F3, FT7, FC3, F4, F8, FC4, FT8, Fz, FCz; posterior: TP7, CP3, P7, P3, CP4, TP8, P4, P8, CPz, Pz). We included the distributional factor of anteriority because the N400 effect often has a central-posterior focus. We also conducted planned comparisons between the nonword and real word conditions within each level of task in order to provide more direct comparisons with the previous MEG findings. Greenhouse-Geisser corrected *p* values are reported when appropriate for tests involving factors with > 2 levels. Because we are interested in the contrasts between conditions, rather than raw ERP responses to individual conditions, we do not report main effects of anteriority.

## Results

### Behavioral data

**Fig 2:**
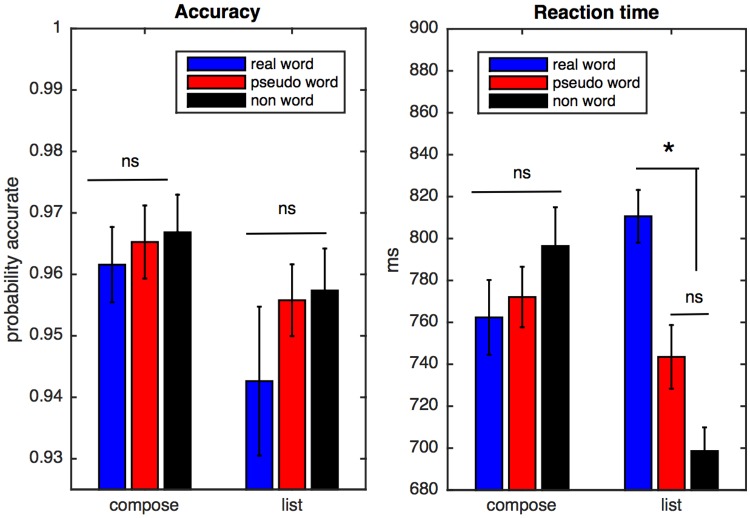
Behavioral data. Accuracy is shown in the left panel, and reaction time in the right panel. A significant effect of task was observed for accuracy, and a significant effect of condition and a significant interaction between task and condition was observed for reaction time.

Behavioral accuracy was high across tasks and stimulus conditions (grand mean = 96%; condition means 94%-97%). Accuracy and reaction time data were submitted to repeated-measures 3 × 2 ANOVAs (condition × task). For accuracy, there was a significant main effect of task (*F*(1, 18) = 6.11, *p* < 0.05), with accuracy being somewhat higher overall in the compose task. For reaction time, there was a significant effect of condition (*F*(2, 36) = 3.43, *p* < 0.05), and a significant interaction between task and condition (*F*(2, 36) = 6.12, *p* < 0.01). Follow up paired *t*-tests, corrected for multiple comparisons, were conducted within task to determine if there were any differences across conditions. No pairwise comparison achieved significance for accuracy data. For reaction time data, within the list task, there was a significant difference between real word and pseudoword conditions, (*p* < 0.001) and between real word and nonword conditions (*p* < 0.0001). Fig 2 shows the behavioral data. These results differ somewhat from those reported in [1], which reported higher reaction times for the list task, and no significant effect of task on accuracy. However, like the behavioral results reported in [1] these results show a processing advantage in the compose task overall, as participants were significantly more accurate in this task.

### Electrophysiological data

**Fig 3:**
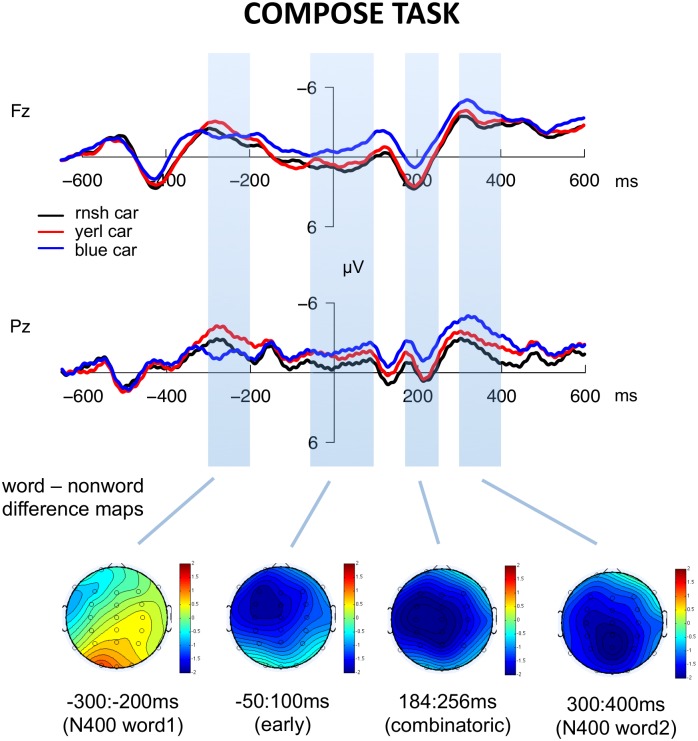
ERP waveforms and topographical distributions for the three conditions in the compose task. Waveforms illustrate the responses at representative electrodes Fz and Pz. Scalp maps illustrate the estimated activity resulting from the subtraction real word—nonword (*blue car*—*rnsh car*) averaged across the time-window indicated.

**Fig 4:**
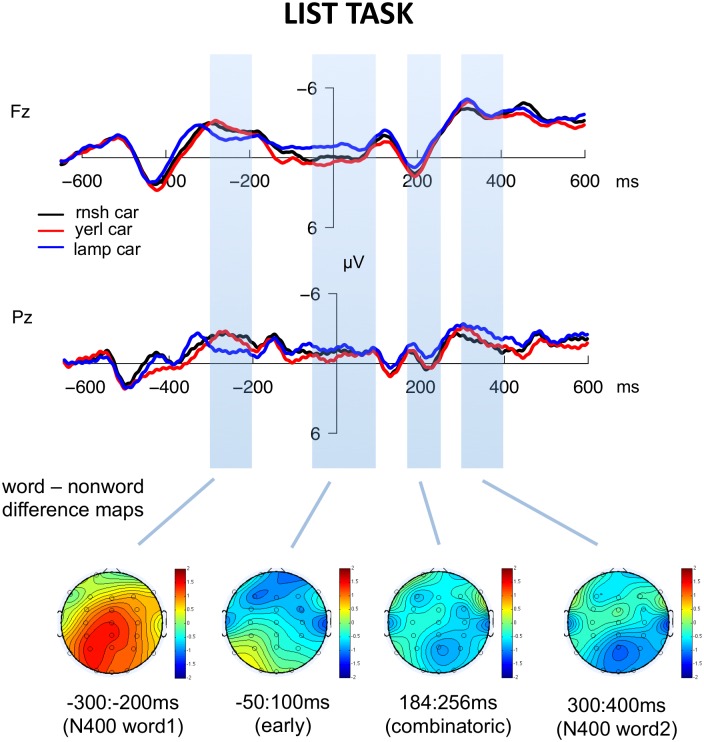
ERP waveforms and topographical distributions for the three conditions in the list task. Waveforms illustrate the responses at representative electrodes Fz and Pz. Scalp maps illustrate the estimated activity resulting from the subtraction real word—nonword (*lamp car*—*rnsh car*) averaged across the time-window indicated.

**Fig 5:**
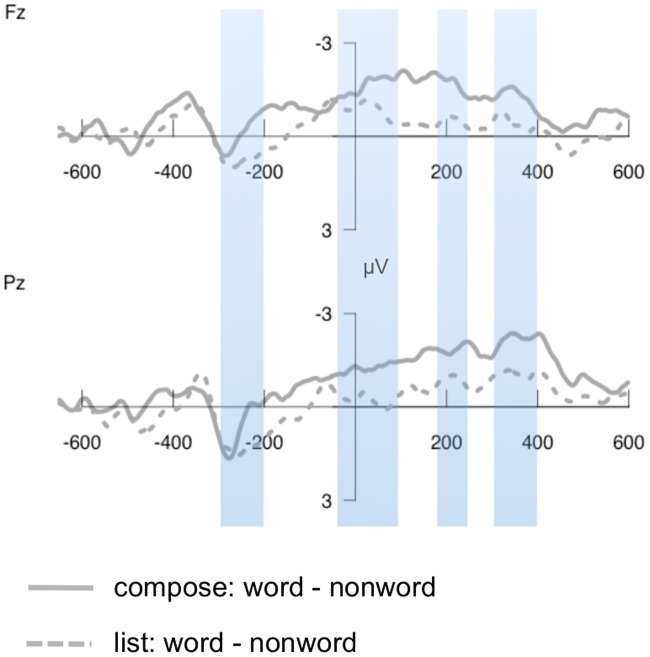
ERP difference waveforms. The upper panel shows the difference between word and nonword responses (word—nonword) at electrode Fz, and the bottom panel shows the difference at electrode Pz.

Figs 3 and 4 illustrate the ERPs to each condition at several representative electrodes; ERPs across all electrodes are included in S1 Fig (compose task) and S2 Fig (list task). Visual inspection indicates several apparent differences due to combinatorial context: in the compose task only, an early negativity for the real word condition relative to the other conditions over anterior electrodes, which appeared to start immediately prior to the presentation of the second word; and a more broadly distributed pattern of increased negativity for the real word condition starting at around 150ms after the presentation of the second word. Fig 5 illustrates the real word—nonword difference waves for both tasks in order for more direct comparison. Below we report statistical analyses that assess the reliability of these differences.

### N400 time-window, word 1 (-300:-200ms)

We conducted analyses in the N400 window following word 1 (-300: -200ms of the epoch). Pseudowords and nonwords showed more negative N400 amplitudes than real words in both tasks. The omnibus ANOVA thus demonstrated significant effects of condition (*F*(2, 36) = 7.6, *p* <.01), and an interaction between condition and anteriority (*F*(2, 36) = 4.7, *p* < .05).

#### Combinatorial time-window (184:256ms)

Visual inspection showed real words eliciting greater negativity than nonwords in the compose task across virtually all electrode sites, with little difference between conditions observed in the list task. The omnibus ANOVA revealed a significant main effect of condition (*F*(2, 36) = 5.3, *p* < 0.01), and an interaction between task and anteriority (*F*(1, 18) = 4.8, *p* < 0.05), however, the interaction between task and condition did not reach significance (*F*(1, 18) = 1.8, *p* = .18). We conducted subsequent planned comparisons focusing on the difference between the real word condition and the nonword condition in each task. In the compose task, the real word condition showed a broadly distributed negativity relative to the nonword condition (*F*(1, 18) = 12.2, *p* < .01), while no effect was observed in the list task (*F*(1, 18) = .9, *p* = .3).

To assess whether initial combinatorial processes are gated by phonological well-formedness, we also conducted comparisons in each task between the nonword and the pseudoword conditions. Visual inspection suggested little difference between these conditions in the compose task, and a small increased frontal negativity for nonwords relative to pseudowords in the list task. Statistical analyses demonstrated no significant differences between nonwords and pseudowords in this time window in either task (*p*s > .2).

#### N400 time-window, word 2 (300:400ms)

A pattern similar to that in the combinatorial time window can be observed here. A broad negativity elicited by real words compared to nonwords was seen in the compose task, while the opposite is seen in the list task. The omnibus ANOVA demonstrated a significant main effect of condition (*F*(2, 36) = 4.8, *p* < 0.05), and a significant interaction between task and anteriority (*F*(1, 18) = 5.5, *p* < 0.05), but the interaction between condition and task was not significant (*F*(1, 18) = 1.1, *p* = .35). However, as in the combinatorial time-window, in subsequent planned comparisons, the real word condition in the compose task showed a broadly distributed negativity relative to the nonword condition (*F*(1, 18) = 9.3, *p* < .01), while no significant differences were observed in the list task (*F*(1, 18) = 1.1, *p* = .3).

#### Early time-window (-50-100 ms)

Immediately prior to the onset of the second (critical) word in the compose task, visual inspection suggested a negative shift over frontal-central electrodes for the real word condition relative to the pseudoword and nonword conditions which continued into the first 100ms after critical word onset. The omnibus ANOVA for this time period revealed a significant main effect of condition (*F*(2, 36) = 4.6, *p* < 0.05) and an interaction between condition and anteriority (*F*(2, 36) = 4.1, *p* < .05), but the interaction between condition and task did not reach significance (*F*(2, 36) = 1.6, *p* = .2). However, planned comparisons between the real word and nonword condition in each task separately demonstrated a robust main effect of condition in the compose task (*F*(1, 18) = 9.5, *p* < .01), with little difference observed in the list task (*F*(1, 18) = .9, *p* = .4).

#### N400 effect localizer

In order to subjectively assess the similarity in topographical distribution of the combinatorial effects to standard N400 effects, we examined data from a contrast between predictable and unpredictable adjective-noun pairs from a subset of 12 participants. To confirm that these participants demonstrated reliable N400 effects of predictability, we conducted a repeated-measures 2 × 2 ANOVA (predictability × anteriority) on the same 20 electrodes and the same 300:400ms time-window as the main experiment. We observed robust effects of predictability on N400 amplitude in the N400 localizer during this 300:400ms time-window. The ANOVA revealed a significant main effect of condition (*F*(1, 11) = 22.5, *p* < .01). Despite the common posterior focus of the N400 effect, the interaction between condition and anteriority was not significant (*F*(1, 11) = .65, *p* > .1).

**Fig 6:**
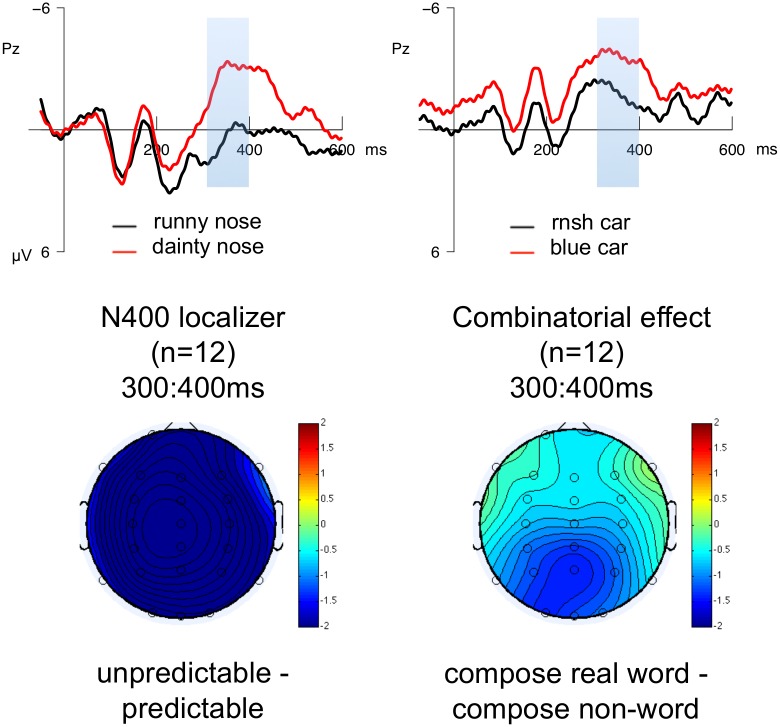
Comparison of ERPs for N400 localizer and main experiment. The upper panels show the waveforms and, and the bottom panels show the topographical distributions for the N400 localizer experiment and the combinatorial effect in the compose task, for the 12 participants who participated in both experiments. Waveforms illustrate the responses at representative electrode Pz. Scalp maps illustrate the estimated activity resulting from the subtractions (unpredictable—predictable) and (real word—nonword) in the 300:400ms time-window.

Fig 6 illustrates the comparison of the combinatorial effect and the N400 localizer effect in the subset of 12 participants who had data from both paradigms. The timing and distribution of these effects was somewhat similar, although the combinatorial effect appeared to have a slightly more anterior distribution. In order to assess whether the effects of predictability (as assessed by the N400 localizer) and combinatoriality (as assessed in the main experiment) in the 300:400ms time-window differed from each other in anterior-posterior distribution, we conducted within-subjects analyses across experiments on the subset of 12 participants who had data from both paradigms. In order to create a common factor for contrasting the ‘combinatorial’ and the ‘predictability’ effects, the combinatorial real word condition and the low predictability condition in the localizer were labelled as ‘high integration effort’ and the nonword condition and the high predictability condition were labeled as ‘low integration effort’. We then conducted a repeated-measures 2 × 2 × 2 ANOVA (experiment × integration effort × anteriority) that included data from both the predictability comparison in the N400 localizer (e.g., *runny nose* vs. *dainty nose*) and the nonword vs. real word comparison in the compose task (e.g., *rnsh car* vs. *blue car*). Because we did not have a priori hypotheses about how raw N400 amplitudes or the size of N400 differences should differ across combinatorial and predictability manipulations, we did not evaluate simple effects of experiment and focused only on the presence or absence of a 3-way interaction between experiment, integration effort, and anteriority. The results of this analysis did not provide clear evidence of reliable differences in distribution, as the 3-way interaction between experiment, integration difficulty, and anteriority did not reach significance (*F*(1, 11) = 1.1, *p* = .32).

## General Discussion

The goal of the current study was to investigate the ERP correlates of basic linguistic combinatorial operations using an extended version of Bemis and Pylkkänen’s [1] MEG paradigm. We report three main findings. First, we observed a qualitatively similar pattern to Bemis and Pylkkänen [1] between 184:256ms, which in EEG this difference took the form of an increased centro-parietal negativity for combinatorial conditions relative to non-combinatorial conditions in the compose task. Second, we found that in EEG this increased centro-parietal negativity continued through 400ms post-noun-onset, and that this effect appeared somewhat similar in timing and distribution to N400 effects of predictability measured in the same participants. Taken together with the localization results from Bemis and Pylkkänen [1], these data suggest that the semantic composition process may modulate either the same neural generators that are responsible for standard N400 effects or closely neighboring ones. Finally, we observed initial evidence that activity in combinatorial trials began to be more negative than the comparable nonword control immediately *preceding* the onset of the critical noun (-50:100ms). If substantiated in subsequent work, the timing of this effect would indicate an anticipatory process associated with combination, such as the initialization of a structural frame.

Before moving into our discussion of the results, it is worth noting two concerns with regard to our design, shared with that of Bemis and Pylkkänen [1]. First, the real words are parts of the English lexicon, while pseudowords and nonwords are not. This implies that the former can be combined with other words, while the latter cannot. The differences observed across the conditions could thus be attributable to two sources: whether the first stimulus is a real word, or whether the first stimulus can combine with the second one. This confound is shared with many other studies which, for instance, contrast sentences with real words versus sentences that use so-called “jabberwocky” stimuli (modeled on Lewis Carroll’s Jabberwocky poem, which contained nonwords in English-like structures: ‘*twas brillig, and the slithy toves/did gyre and gimble in the wabe*). However, if the contrast is driven by the lexical status of the first word, then we would expect *cup car* and *blue car* to pattern together, and *rnsh car* and *yerl car* to pattern together. This is not what we observe; *cup car* patterns with the nonwords and pseudowords, and *blue boat* is distinct from all the other conditions.

It is also possible that some participants were composing the pairs of nouns in the list task. Some of the nouns in our materials form lexicalized compounds (e.g., boat house), and other pairs may have been easily compounded with one another (e.g., plane bag, which might be the sort of bag you bring on an airplane). Again, this was also a property of the original Bemis and Pylkkänen study that we were attempting to replicate [1]. But more importantly, we think that this is unlikely to have occurred in the current experimental context for several reasons. If participants really were composing the noun pairs in the list task, one might predict very low response accuracies for this condition since there were no pictures of the unintended compounds in the experiment such as boat houses, let alone some of the more improbable compounds (e.g., *cross cup, flag bone, shoe bow*); if participants were reliably composing these possible compounds, they should have answered ‘no match’ for the images they saw on the screen. However, we excluded all participants with response accuracies lower than 70% for any condition, meaning that any participants who used this strategy would have been excluded. The nature of the task also strongly discouraged the formation of noun-noun compounds, given that the instructions to the task indicated that listeners should try to consider each word in isolation.

### 184:256ms: Binding lexical semantics to syntactic structure

In the composition task, we observed increased negativity in the combinatorial condition relative to the nonword condition with a centro-posterior, slightly leftward distribution in the same time window that Bemis and Pylkkänen [1] report increased MEG responses to combination in the left anterior temporal lobe (LATL). Like Bemis and Pylkkänen [1], we did not observe significant differences in this time-window for the list task, although in our dataset the interaction between task and condition did not reach significance.

Bemis and Pylkkänen [1] discussed the possibility that this time-window reflects combinatorial syntactic operations, but subsequent work from their group appears more consistent with a semantic locus for the effect (e.g., [19, 20, 33])), consistent with prior work implicating the ATL in semantic memory representation [34, 35]. For example, Westerlund and Pylkkänen [19] show that increased ATL activity in combinatorial contexts is observed when adjectives are combined with less conceptually specific nouns like *blue boat*, but not when adjectives are combined with more specific nouns like *blue canoe*.

Although the current results demonstrate that the basic combinatorial effect in this time-window replicates as a centro-posterior negativity in EEG, our use of Bemis and Pylkkänen’s [1] original design does not allow us to derive insights about the specific mechanisms generating this early response. However, based on previous top-down parsing models and the earlier differences we observe between conditions in the -50:100ms time-window, we tentatively hypothesize that the combinatorial effects observed between 175-250ms correspond to the binding of a lexical item into an empty structural position that has been pre-generated in the -50:100ms time window. One piece of evidence in support of this idea is that Bemis and Pylkkänen [1] also reported combinatorial effects in the superior parietal lobule (SPL) in the same time-window. As they note, outside of the domain of language SPL has been argued to play an important role in binding visual features [36] and manipulating objects in short-term memory [37], analogously to the working memory manipulation required to bind a word into a structure. Simultaneous ATL-SPL activity might therefore represent lexical semantic features being drawn from the lexicon and bound into the pre-generated syntactic object. This is speculative, however, particularly because to our knowledge SPL has not been associated with syntactic processes in previous work on neuroanatomy of language.

### 300:400ms: Semantic integration/iterative lexical access

In the 300-400ms time-window following the critical noun we continued to observe a broadly-distributed increased negativity for the combinatorial condition relative to other conditions. This effect overlapped with the time-window in which N400 effects are typically observed [21]. Its topographical distribution was not clearly distinct from the distribution of the N400-like effects observed to the first word when comparing pseudowords and nonwords with adjectives, corresponding to N400-like effects found in previous experiments related to pseudowords and nonwords. Its distribution was also somewhat similar to the distribution of the lexical predictability N400 effect obtained in a subset of our participants. As such, these data are consistent with the hypothesis that the combinatorial effect in this time-window is an N400 effect analogous to those found in numerous other linguistic experiments. Although further work is needed to establish this point more conclusively given the limited spatial resolution of EEG, in what follows we consider how combinatorial context could modulate the N400 according to major accounts of the N400 effect.

The directionality of the current combinatorial effect might at first appear to contradict the generalization that N400 amplitude is often smaller in supportive contexts. The ‘integration’ account of the N400 states that this component reflects the computations that combine or ‘integrate’ the current word into the ongoing representation of the local semantic context [38–41]. When a word is strongly predicted in a sentence or discourse context, the N400 response to that word is reduced, which is understood on this view as reflecting ‘easier’ contextual integration [42]. Some authors have also observed a more negative N400 response when semantic coercion or type-shifting is required (e.g., in cases where the author finished the book means that the author has finished reading the book), which is also consistent with the idea that the N400 reflects semantic integration [43, 44]). In the current study, the increased negativity to concrete nouns preceded by color adjectives observed between 300:400ms could therefore be understood as an increased N400 reflecting semantic combinatorial operations, a claim which has precedence in the literature for sub-sentential morphosyntactic structure [45–47]. In the nonword and pseudoword conditions, semantic composition is simply impossible (i.e., *rnsh* and *yerl* have no semantic content to create a context for integration, so the effect is smaller). The list task discouraged semantic composition in the real word condition; constructing a semantic representation of *cup car* as a car with some of the attributes of a cup is not conducive to the performance of the picture-matching task (i.e., the subject was looking for a match to be a picture of a cup *or* a picture of a car). The real word condition in the composition task is the only condition in which semantic composition is either possible or not counterproductive to the performance of the task, and so it seems possible to align these results with the body of literature that associates the N400 with semantic integration processes.

On the so-called ‘lexical’ or ‘semantic memory’ account of the N400, this component rather reflects the activation/retrieval of lexical or conceptual representations from long-term memory [23, 48–50]. According to this view, in contexts where some word or concept is highly predictable, it is more easily accessible from long-term memory. Differential voltage amplitudes are interpreted as reflecting the ease/difficulty of memory access in that a smaller ‘neighborhood’ of lexical or conceptual representations needs to be activated when the representation best fitting the current stimulus is easily identified. The sensitivity of the N400 to repetition priming and semantic priming manipulations within word lists [51] follows naturally from this view, as well as recent work showing that when directly contrasted in the same experiment, the N400 is much more sensitive to predictability than semantic plausibility [25]. However, it is not immediately obvious how such an account could explain an N400 effect for the combinatorial manipulation in the current study. The word or concept associated with *car* should not be more difficult to retrieve from the lexicon (as indexed by having a more negative N400 effect) having just retrieved *blue* than in contexts where the lexicon has not been accessed at all (*rnsh*, *yerl*). This is particularly true since to the extent that any differences exist in the predictability of *car* across conditions, it should be more predicted after *blue* (presuming that the reader has ever encountered the phrase *blue car* outside the experiment). A priming account, however, would predict the opposite polarity difference: in priming experiments, one would expect a reduced negativity on *car* when preceded by *blue*, compared to when it is preceded by *rnsh*. The directionality of the effect reported here is precisely the opposite.

However, we believe that there is also a natural way in which the ‘semantic memory’ view of the N400 can account for ‘combinatorial’ N400 effects. Following the lead of previous researchers [8, 21, 52, 53], the intuition we share is that combinatorial operations and semantic memory access operations are not sequential processes but are tightly interwoven and highly interdependent. Therefore, while N400 amplitude might proximally reflect the activation of stored memory representations, the combinatorial demands of the context impacts what is activated and therefore indirectly modulates the N400. Humphries et al. [9], following Barsalou [54], point out that in the sentence *The shipwreck victim survived by clinging to a basketball* the meaning of *basketball* takes on a detail which may be absent in isolation: basketballs are buoyant. Deriving a complete semantic interpretation of the sentence requires accessing this knowledge about basketballs, but this knowledge probably would not have been retrieved if *basketball* had been encountered in isolation. Thus, the local syntactic and semantic context motivates the retrieval of increasingly fine-grained or specific information from semantic memory [53]. For example, the semantics of color adjectives are more complex than at first glance: a blue pen, a blue house, and a blue car are not all blue in the same way, as different hues of blue are canonically associated with these objects and color modifiers are canonically taken to apply to different aspects of these objects (the ink of the pen vs. the external surface of the car). Therefore, in our experiment, such effects could be due to re-access and refinement of either the semantic information of the color adjective or the concrete noun.

On either account, we should note that is still not completely straightforward to relate the interpretation of the 300-400ms difference as an N400 effect to the localization results reported by Bemis and Pylkkänen [1]. Although they suggested that activity in this time-window reflected combinatorial semantic operations, they localized this MEG activity to the ventromedial prefrontal cortex, where their group has previously localized effects of semantic coercion [55] and semantic mismatch [56]. However, the world knowledge violations that are thought to elicit N400 effects on the integration view were not localized to vmPFC [56] and neither has this been the case for any other N400 study, to our knowledge. The same is true for the angular gyrus region that demonstrated combinatorial effects in this time-window in their later study [17]. Therefore, one possibility is that despite the surface similarity, our 300-400ms effect is not a standard N400 effect but is rather a combinatorial semantic response generated by a different area or set of areas, such as the vmPFC or angular gyrus. This would explain why the topographical distribution of the combinatorial effect was not exactly identical to that of N400 effects of predictability and lexicality.

However, it is also the case that Bemis and Pylkkänen [1] report a non-significant combinatorial effect in left anterior temporal cortex in the 350-400ms time-window. If reliable, this would be highly consistent with the hypothesis that the differences in this time-window are standard N400 effects, as N400 effects have been localized to left ATL in studies using a variety of imaging measures [57–62]. Paralleling the two accounts of the N400, an ongoing puzzle has been whether left ATL is involved in basic long-term semantic memory storage or combinatorial processing, as damage to ATL seems to result in fundamental semantic memory deficits (see [35] for review) but left ATL activity increases for materials that allow combinatorial processing, such as sentences when compared to lists [4, 6, 9, 16]. However, Wilson et al. [53] show that basic syntactic operations seem to be preserved in patients with ATL damage and, as discussed above, suggest that a means for reconciling these results is to assume that more extensive and frequent access of semantic memory in left ATL is required to interpret words in sentence contexts than words in isolation.

### Early Negativity: -50:100ms

Finally, we observed preliminary evidence for a heretofore unreported combinatorial effect immediately preceding and following the onset of the critical second word. In the data reported here, the real word condition in the compose task (e.g., *blue car*) elicited significantly greater negativity than the nonword or pseudoword conditions beginning approximately 50 ms before the presentation of the target word. Reliable differences were not observed in this time-window for the list conditions. Because context words were reasonably well-matched across tasks and ERP responses to the context word appeared to reconverge before diverging shortly before the critical word, this effect seems most likely to reflect an anticipatory process associated with combinatorial activity. However, we note that this analysis was done post-hoc based on visual inspection and that the interaction between condition and task did not reach significance, and for this reason the interpretation that follows must remain tentative until subsequent replication.

We hypothesize that this early difference specifically corresponds to anticipatory syntactic structure initialization, which has been proposed by many previous authors to be a key property of sentence processing [63–69]. On this view the adjective-noun syntactic structure is built *before* the presentation of the noun, with an empty slot where the (inevitable) noun will go, and upon the noun’s visual presentation and its lexical retrieval it fills the prepared slot. Differences in activity immediately prior to the noun in the combinatorial condition would therefore reflect the processes involved in predictively generating this connected phrase structure. We note that a recent MEG study has reported an analogous neural measure of lexical prediction processes immediately prior to critical word presentation [70].

We suggest that the early effect we observe here is a reflex of syntactic structure initialization rather than a more general predictive mechanism (e.g., computing the probability of the lexical category of the upcoming stimulus, or initializing a new open slot in generic short-term verbal memory) because the fact that the second stimulus will be a noun is completely and equally predictable across all tasks and conditions in the experiment. Another possibility is that the early effect we see is the result of a lexical prediction generated by the color adjective, rather than a syntactic prediction. For example, *red* presumably precedes some words (e.g. *rose* or *blood*) more frequently than others in real world contexts, and thus the difference across conditions might be attributable to the generation of a specific lexical prediction after adjectives but not after nouns, nonwords or pseudowords. However, it is worth noting that nouns are also followed by some words more than others and thus might be expected to generate automatic lexical predictions to the same extent as adjectives. On the other hand, if lexical prediction were sensitive to the experimental context, these results would also be unexpected because in the current experiment the limited set of critical nouns appeared with equal probability following all color adjectives. Further work would be needed to disentangle these possibilities.

One important question is why previous MEG studies using the same paradigm (e.g., [1, 17]) did not report this early effect. We believe the most likely explanation is that these studies used the default procedure of baselining the responses to the time-window immediately preceding the critical second word of the trial. Since the effect we observed began exactly in this time-window, baselining the responses across conditions would naturally act to cancel this effect. However, further replication is an important next step. If borne out by further work, the early effect observed here could potentially provide a useful new measure for investigating predictive structure building.

## Conclusion

This study aimed to describe the electrophysiological correlates of simple adjective-noun composition. We replicated Bemis and Pylkkänen’s [1] MEG observation of differential responses to words in combinatorial and non-combinatorial contexts between ∼184-256ms, and we showed that this effect has a centro-posterior distribution in EEG. We tentatively hypothesize that this ‘combinatory’ effect might be associated with binding lexical material into an empty syntactic slot. We also found that the difference between combinatorial and non-combinatorial conditions between 300:400ms bears some similarities to classic N400 effects in distribution and latency, and we have indicated how this could be reconciled with the both prior localization results and with competing accounts of the N400. Finally, we observed preliminary evidence for a hitherto unreported pre-stimulus effect on the noun, which we suggest may be the reflex of a predictive parsing mechanism: the initialization of empty syntactic structure. Together, these results provide new insights into the neural computations supporting basic phrase-building, and suggest that ERPs can be profitably used alongside MEG to investigate these processes in future work.

## Supporting Information

S1 Fig ERP waveforms in the compose task. The event-related potentials recorded at each electrode in the compose task. Voltage timeseries for the real word condition are plotted in blue, for the pseudoword condition in red, and for the nonword condition, in black.
(TIFF)

S2 Fig ERP waveforms in the list task. The event-related potentials recorded at each electrode in the compose task. Voltage timeseries for the real word condition are plotted in blue, for the pseudoword condition in red, and for the nonword condition, in black.
(TIFF)

S1 File README. This file contains information describing the structure of the data contained in S4-S5.
(TXT)

S2 File S2_File.zip. This file contains the event-related potentials recorded in the N400 localizer task.
(ZIP)

S3 File S3_File.zip. This file contains the event-related potentials recorded in the main experiment.
(ZIP)
