# Supplementary figures and images for: The Electrophysiology of Basic Phrase Building
Source: PLoS One. 2016 Oct 6;11(10):e0158446. doi: 10.1371/journal.pone.0158446 (PMC5053407; doi:10.1371/journal.pone.0158446)

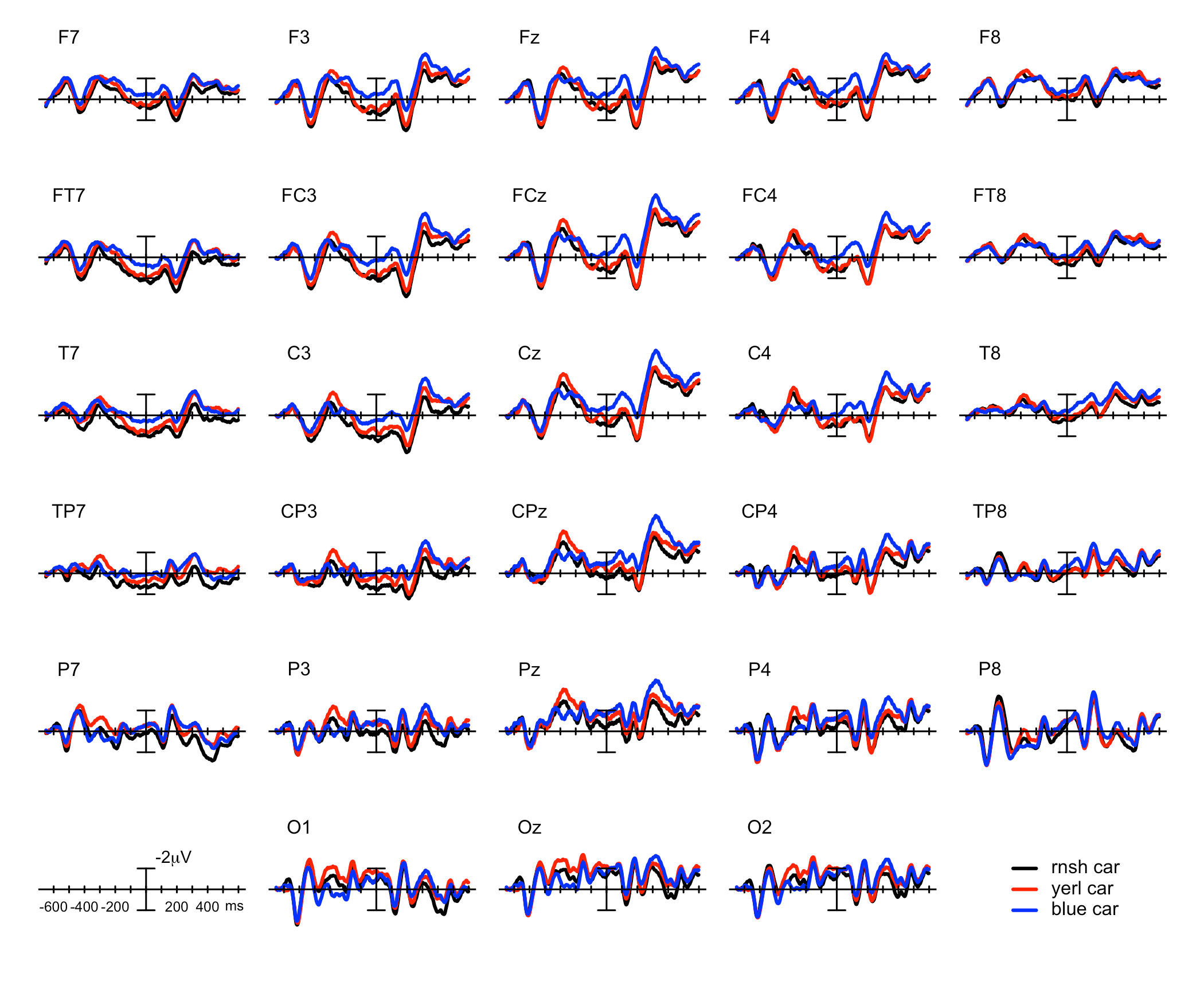

Supplement: S1 Fig — The event-related potentials recorded at each electrode in the compose task. Voltage timeseries for the real word condition are plotted in blue, for the pseudoword condition in red, and for the nonword condition, in black. (TIFF) [file pone.0158446.s001.tiff]
